# First Case of ‘Soft Flesh’ Induced by *Kudoa thyrsites* in an Atlantic Bonito ( *Sarda sarda* )

**DOI:** 10.1111/jfd.14053

**Published:** 2024-12-03

**Authors:** Felice Panebianco, Stefano Bagatella, Tiziana Civera, Selene Rubiola

**Affiliations:** ^1^ Department of Veterinary Sciences University of Turin Turin Italy

**Keywords:** Atlantic bonito, *Kudoa thyrsites*, post‐mortem myoliquefaction, soft flesh

## 1 Introduction

The Atlantic bonito, *Sarda sarda* (Bloch 1793), is one of the most exploited scombrid in the Mediterranean Sea (Campo et al. 2006), where it constitutes a commercially valuable fish mainly targeted by artisanal fisheries (Ollé‐Vilanova et al. 2024). Cases of post‐mortem myoliquefactive kudoosis, also known as *Kudoa*‐induced ‘soft flesh’ or ‘milky flesh’ have been reported in some fish species from the Mediterranean Sea, including one swordfish ( *Xiphias gladius* ) (Gaglio et al. 2010), two gobiid fish (*Pamatoschistus minutus* and *P. micrus*) (Pampoulie et al. 1999) and two silver scabbardfish ( *Lepidopus caudatus* ) (Giulietti et al. 2019), rendering the fish unmarketable.

The genus *Kudoa* (Meglitsch 1947) includes over 100 myxozoan histozoic parasite species (Cnidaria: Myxozoa: Myxosporea: Multivalvulida) infecting marine and estuarine fishes, whose myxospores are morphologically characterised by 4–13 shell valves and polar capsules. *Kudoa* spp. are found mainly in the skeletal muscle of fish, although they can also affect other organs (Moran, Whitaker, and Kent 1999). Despite usually being not pathogenic for the hosts, some species negatively impact the quality of fishery products due to the formation of macroscopic cysts in affected tissues or post‐mortem myoliquefactive autolysis. This phenomenon, caused by the release of proteolytic enzymes, occurs up to 48 h after death, thereby often going unnoticed during quality controls in the fishing industry. Thus, affected fish can reach retailers or final consumers and be discarded, leading to economic losses for the seafood sector (Giulietti et al. 2024). Additionally, some *Kudoa* spp. can cause foodborne diseases manifesting with gastrointestinal symptoms after the consumption of raw fish (Inoue et al. 2024). A *Kudoa* species frequently associated with ‘soft‐flesh’ is *Kudoa thyrsites* (Gilchrist 1924), characterised by stellate/cruciform myxospores with unequal spore valves and polar capsules. *Kudoa thyrsites* has been reported to infect many marine teleosts worldwide, including different economically valuable fishes (Whipps and Kent 2006; Giulietti et al. 2024).

Here, we report the first case of *Kudoa thyrsites*‐induced post‐mortem myoliquefaction in an Atlantic bonito caught in the Mediterranean Sea.

## 2 Case Presentation

In March 2024, a consumer purchased two Atlantic bonitos from a retailer in Livorno (Tuscany, Italy). The fishes belonged to the same batch and were caught in FAO Subarea 37.1. At home, the consumer noticed that one specimen exhibited a soft texture, with a slight indentation of the skin following gentle pressure. After filleting, the musculature appeared soft, whitish and exudative. In contrast, the musculature of the other bonito showed normal appearance. The altered specimen was frozen at −20°C and sent to the University of Turin for investigation.

Upon arrival, the specimen was thawed for 12 h at 4°C. To assess the presence of myxospores, 3 × 3 × 3 mm muscle fragments were excised with forceps and pressed onto glass slides to obtain up to 10 impression smears per slide. Smears were covered with room‐temperature phosphate‐buffered saline (PBS) and then with a glass coverslip to avoid drying. Slides were observed under a light microscope at 100× magnification with oil immersion (Nikon Eclipse E200, Japan). Multiple images were captured with LAS X Core software (Leica Microsystems) and measurements of unfixed spores on apical (*n* = 10) and side view (*n* = 5) were assessed according to Burger and Adlard (2010a). For histological evaluation, sections of muscles were immersion‐fixed overnight in 10% neutral buffered formalin and subsequently embedded in paraffin. Four‐micrometre‐thick tissue slices were then sectioned with a microtome and stained with haematoxylin and eosin (HE) or Giemsa. Slides were observed with a Leica DM 750 microscope coupled with a Leica ICC50 W camera and pictures were captured with the Leica Microsystem LAS EZ software.

**FIGURE 1:**
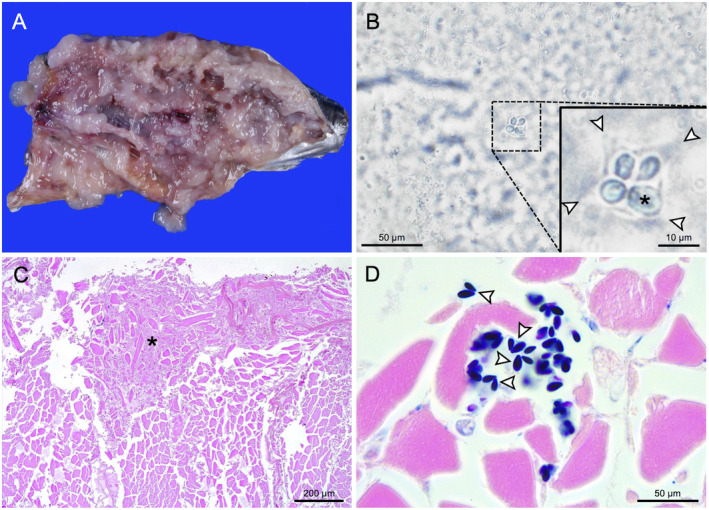
Gross and histological aspects of muscular kudoosis in an Atlantic bonito. (A) Macroscopic appearance of the musculature, showing severe softening and myoliquefaction. (B) Apical view of a myxospore, consisting of four distinct polar capsules (the largest one is indicated with an asterisk), each surrounded by shell valves (arrowheads), detected through light microscopy on impression smears from unfixed musculature (100× magnification). (C) Extensive area of myoliquefaction with degenerated myofibers and amorphous debris (asterisk) overlying an area of muscle with preservation of tissue architecture (4× magnification, HE). (D) Myxospores closely associated with fragmented myofibers and free in the muscular interstitium. Notice the dark blue polar capsules and the pyramidal aspect of myxospores on side view (arrowheads) (100× magnification, Giemsa).

On external examination, the musculature showed a diffuse decrease in consistency upon palpation. On cut section, both epaxial and hypaxial musculature displayed diffuse, severe softening, ranging from gelatinous consistency to frank liquefaction (Figure 1A). Impression smears of unfixed muscle revealed the presence of a moderate number of myxospores which, on apical view, displayed a stellate appearance with four shell valves, each containing an oval to drop‐shaped polar capsule, one of which appeared larger than the others (Figure 1B). On side view, the spores appeared pyramid‐shaped with the narrow end of the polar capsule oriented towards the apex. Measurements of myxospores from unfixed smears are indicated in Table 1. Histological examination revealed extensive, multifocal areas of myofiber degeneration and myoliquefaction, consisting of amorphous to finely granular, eosinophilic debris intermixed with fragmented and/or swollen, hypereosinophilic myofibers (Figure 1C). No associated inflammatory reaction was present. Multiple small aggregates of myxospores, displaying a morphology consistent with that of unfixed myxospores, were observed in association with fragmented myofibers or within the muscular interstitium (Figure 1D). No intact pseudocysts were detected, likely due to the liberation of myxospores from sarcoplasmic pseudocysts following the myoliquefactive process in conjunction with freezing/thawing.

**TABLE 1:** Morphological measurements (expressed in μm) of myxospores from unfixed impression smears of muscle from an Atlantic bonito (according to Burger and Adlard 2010a).

	Mean ± SD (range)
Width (apical view)	17.2 ± 1.2 (15.4–19.4)
Thickness (apical view)	11.8 ± 0.7 (10.8–13.4)
Length (side view)	8.5 ± 1.3 (7.5–10.6)
Apical large polar capsule length (apical view)	7.9 ± 0.9 (6.2–9.6)
Apical large polar capsule width (apical view)	6.3 ± 0.8 (4.4–6.9)
Apical small polar capsule width (apical view)	4.2 ± 0.3 (3.4–4.5)
Side large polar capsule length (side view)	7.6 ± 1.0 (6.7–9.2)
Side small polar capsule length (side view)	5.2 ± 0.3 (4.8–5.6)

For the molecular identification of the myxosporidia, two 25‐mg aliquots of liquefied muscle were collected; DNA was extracted using the DNeasy Blood & Tissue Kit (QIAGEN, Hilden, Germany) following the tissue protocol. A ~530‐bp fragment of the 18S ribosomal RNA gene (18S rRNA) and a ~770 bp fragment of the 28S ribosomal RNA gene (28S rRNA) were amplified using the *Kudoa* general primer pairs 18e/KUD6R (Hillis and Dixon 1991; Whipps et al. 2003) and Kt28S1F/28S1R (Burger and Adlard 2010b; Whipps et al. 2004), respectively, as described by Meng et al. (2011). PCR products were purified applying the ExoSAP‐IT purification kit, sequenced using the v1.1 BigDye Terminator Ready Reaction Kit (Applied Biosystems by Thermo Fisher Scientific, USA) and run on an Applied Biosystems SeqStudio Genetic Analyzer (Thermo Fisher Scientific, Waltham, MA). Forward and reverse sequences were assembled into consensus sequences and aligned using MEGA 11 (Kumar et al. 2018). The identity of each sequence was checked using the BLASTn sequence similarity search against the NCBI nucleotide database.

Phylogenetic analyses were conducted separately. For the 28S rRNA gene, 23 partial sequences were used, including one of the two identical sequences generated in the present study and 22 *Kudoa* spp. sequences retrieved from GenBank based on the BLAST identity score, spore morphology (cruciform myxospores) and geographic distribution; for the 18S rRNA gene, 25 partial sequences were used, including one of the two sequences generated in the present study and 24 *Kudoa* spp. sequences. A sequence of *Kudoa ogaway* was used as outgroup. Multiple alignments were obtained using the ClustalW algorithm in MEGA 11; sequences were manually trimmed so that all queries started and ended at the same nucleotide positions. The phylogenetic trees were reconstructed using the Neighbor‐Joining algorithm and the phylogeny was tested with the bootstrap method.

**FIGURE 2:**
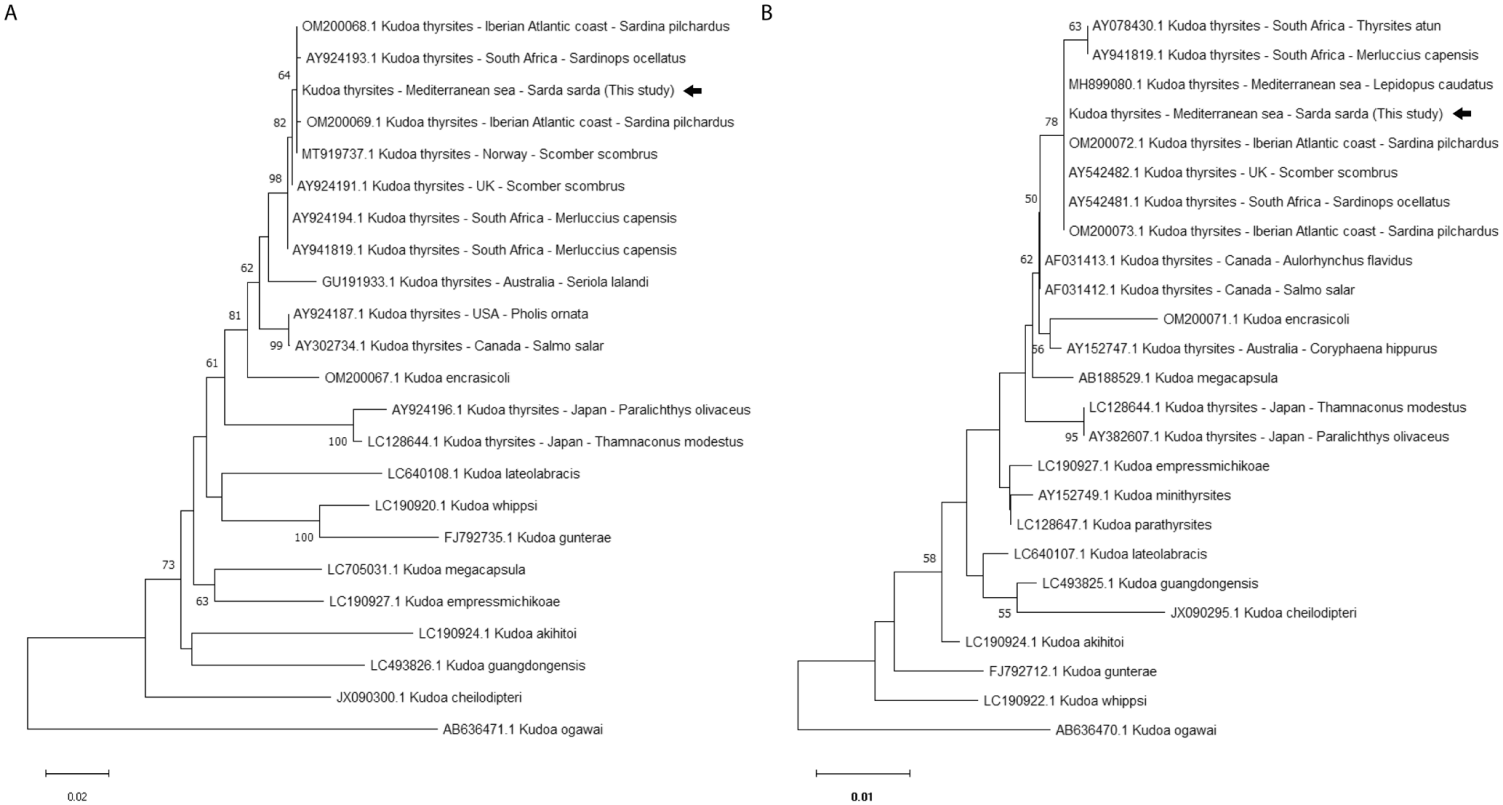
Neighbor‐Joining phylogenetic tree based on the 28S rRNA gene (A) and the 18S rRNA gene partial sequences (B) of the *Kudoa thyrsites* isolate sequenced in the present study (arrows) and of selected *Kudoa* spp. The percentages of replicate trees in which the associated taxa clustered together in the bootstrap test are shown near each node (1000 replications); nodal values lower than 50 are not shown. For *K. thyrsites* isolates, hosts and geographic locations are reported.

Both the 18S rRNA and the 28S rRNA amplification protocols resulted in fragments of the expected size in all liquefied muscle aliquots. The resulting 510 bp 18S rRNA sequences showed 100% identity with each other and 100%–99.6% identity with *Kudoa thyrsites* GenBank entries n. OM200072‐73, AY542481‐82, AY941819, AY078430, AF031412, AY152747 and AF031413 (best match: *K. thyrsites* sequences OM200072‐73 and *K. thyrsites* sequences AY542481‐82) (Iglesias et al. 2022; Whipps and Kent 2006). Similarly, the 662–704 bp 28S rRNA sequences revealed 100% identity with each other and 100%–99.5% identity with *K. thyrsites* GenBank entries n. MT919734‐37, AY941819, OM200068‐69 and AY924191‐94 (best match: *K. thyrsites* sequence MT919737) (Giulietti et al. 2020). The phylogenetic analyses showed close relationships between the *K. thyrsites* sequences generated in this study and those generated from different fish caught in the Atlantic Ocean and from a silver scabbardfish caught in the Mediterranean Sea, which was the sole available *K. thyrsites* sequence generated from a Mediterranean fish so far; conversely, most of the Pacific, Australian and Indo‐Pacific (Japan and South Africa) specimens formed separate subclades (Figure 2) (Giulietti et al. 2020; Iglesias et al. 2022). The sequences generated in the present study were deposited in GenBank (accession numbers PQ246265, PQ246266, PQ246277 and PQ246278).

## 3 Discussion and Conclusions

The occurrence of *Kudoa* spp. in fish muscle poses an emerging challenge for the fishery industry. The primary concerns are centred around economic losses rather than public health, as only a few *Kudoa* species have been associated with food poisoning in humans (EFSA Panel on Biological Hazards 2024; Kang et al. 2020; Kawai et al. 2012; Sung et al. 2023; Suzuki et al. 2015). No cases of foodborne disease have been associated with *K. thyrsites* and *K. islandica*, the two species often associated with post‐mortem myoliquefactive phenomena in fish. However, hypersensitivity reactions to the parasites themselves cannot be excluded (EFSA Panel on Biological Hazards 2024). Therefore, although the risks to consumers appear minimal, the potential pathogenicity of certain species should not be ignored.

Among *Kudoa* spp., *K. thyrsites* is often responsible for muscle alteration in Atlantic mackerel and other species (Giulietti et al. 2022, 2024). To the best of our knowledge, this study presents the first documented case of ‘soft flesh’ caused by *K. thyrsites* in Atlantic bonito. The case involved a highly severe condition, marked by reduced muscle firmness and extensive tissue liquefaction, which became apparent to the consumer immediately after purchasing the fish. Expanding research to study the incidence of these parasites in Atlantic bonito in the Mediterranean Sea is advisable, as this report suggest the spread of *K. thyrsites* within this new host, with a resulting negative impact on the marketability and processing.

The current study also highlights the need to expand our knowledge regarding the occurrence and prevalence of these parasites in other commercially important fish species caught in the Mediterranean Sea. Further research is needed to understand whether climate change, water warming and other environmental variables may influence the incidence of *Kudoa* in fish and the occurrence of ‘soft flesh’ across various fish families.

## Author Contributions

**Felice Panebianco:** conceptualization, writing – original draft, methodology, writing – review and editing, investigation. **Stefano Bagatella:** methodology, software, visualization, writing – review and editing. **Tiziana Civera:** supervision, writing – review and editing, funding acquisition. **Selene Rubiola:** conceptualization, writing – original draft, methodology, investigation, writing – review and editing.

## Consent

All authors have made significant contributions to the present study and agreed to participate. All authors have read and approved this manuscript submission to be considered for publication.

## Conflicts of Interest

The authors declare no conflicts of interest.

## Data Availability

The data that support the findings of this study are openly available in GenBank at https://www.ncbi.nlm.nih.gov/genbank/, reference number PQ246265, PQ246266; PQ246277, PQ246278.
